# Metabolome of human gut microbiome is predictive of host dysbiosis

**DOI:** 10.1186/s13742-015-0084-3

**Published:** 2015-09-14

**Authors:** Peter E. Larsen, Yang Dai

**Affiliations:** 1 Bioengineering Department, University of Illinois at Chicago, 851 South Morgan, SEO218, Chicago, IL 60607 USA; 2 Argonne National Laboratory, Biosciences Division, 9700 South Cass Ave, Argonne, IL 60439 USA

**Keywords:** Dysbiosis, Gut microbiome, Human microbiome, Machine learning, Metabolome modeling, Metagenomics, Microbial communities

## Abstract

**Background:**

Humans live in constant and vital symbiosis with a closely linked bacterial ecosystem called the microbiome, which influences many aspects of human health. When this microbial ecosystem becomes disrupted, the health of the human host can suffer; a condition called dysbiosis. However, the community compositions of human microbiomes also vary dramatically from individual to individual, and over time, making it difficult to uncover the underlying mechanisms linking the microbiome to human health. We propose that a microbiome’s interaction with its human host is not necessarily dependent upon the presence or absence of particular bacterial species, but instead is dependent on its community metabolome; an emergent property of the microbiome.

**Results:**

Using data from a previously published, longitudinal study of microbiome populations of the human gut, we extrapolated information about microbiome community enzyme profiles and metabolome models. Using machine learning techniques, we demonstrated that the aggregate predicted community enzyme function profiles and modeled metabolomes of a microbiome are more predictive of dysbiosis than either observed microbiome community composition or predicted enzyme function profiles.

**Conclusions:**

Specific enzyme functions and metabolites predictive of dysbiosis provide insights into the molecular mechanisms of microbiome–host interactions. The ability to use machine learning to predict dysbiosis from microbiome community interaction data provides a potentially powerful tool for understanding the links between the human microbiome and human health, pointing to potential microbiome-based diagnostics and therapeutic interventions.

**Electronic supplementary material:**

The online version of this article (doi:10.1186/s13742-015-0084-3) contains supplementary material, which is available to authorized users.

## Background

Humans exist, not as individuals, but as superorganisms comprised of human cells that live in an inseparable symbiotic relationship with a vast ecosystem of microorganisms. These human-associated communities are collectively referred to as the human microbiome. Largely invisible, only recent advances in high-throughput sequencing [1–3] have rendered these vital communities observable to scientific research, revealing the importance of the life-long relationships between our microbiome and our health and well-being. The human microbiome provides many crucial services to their human hosts, including defense against colonization by harmful or pathogenic organisms [4, 5], aid in digesting food and provision of essential vitamins and nutrients [6–9], and maintenance of a healthy immune system [10–13]. Conversely, perturbations in these symbiotic communities can have a negative effect on the host’s health, termed dysbiosis [14], which can lead to a variety of human disease states, such as irritable bowel syndrome (IBS) [15–19], autoimmune disorders [20, 21], increased vulnerability to cancers [22, 23], and obesity [24–27]. Dysbiosis of the gut microbiome has been shown to coincide with increased risk of depression [28], and to affect other aspects of the human host’s mental health [29, 30]. Understanding the relationships between human health and the associated microbiome provides a new and valuable tool for diagnostics and potential mechanisms for human therapeutic interventions. Already, microbiome transplants have proven a powerful tool for curing otherwise intractable diseases such as IBS [31–33] or antibiotic resistant *Clostridium difficil*e infections [34, 35]. One mechanism by which the microbiome interacts with its host is through the microbiome’s community metabolism [36–38]. Community metabolism, however, can be independent of community structure [39], making the relationships between the microbiome and host health complex.

Large-scale studies for identifying and characterizing microbiome communities, such as the Metagenomics of the Human Intestinal Tract (MetaHIT) [40] project and the Human Microbiome Project (HMP) [41], have contributed to our understanding of the relationships between microbiome community composition and the host. They have also highlighted that the tremendous diversity of the microbiome presents a significant challenge for analysis of human microbiome data. An individual’s microbiome has a specific community structure, which is defined as the type and relative abundance of all the bacteria present in the microbiome community. A human host’s microbiome is dynamic; changing in response to host behavior, environment, and diet [42–44]. Human microbiomes are also highly divergent from host to host. It has even been proposed that individuals might have unique microbiome community structures [45]. Host environment, diet, and genetics have been implicated in driving this diversity, although many of the variations between human microbiomes remain unexplained. The dynamic nature of these communities impedes our ability to make generalizations applicable across microbiomes.

To leverage the microbiome community for the benefit of human health, analysis approaches will have to explore more than just the community structures of microbiomes to find biologically relevant patterns. It has been reported that relevant patterns do exist and can be found among the highly varied microbiome communities. For example, a study of the microbiomes of a cohort of 4,788 samples taken from 242 adults revealed that although community structures varied, specific metabolic pathways were found across multiple microbiome metagenomes [46]. In another study, it was reported that although the microbiome community structures of individuals and various sampled regions were distinct from one to another, the community structures from one part of the body of an individual were predictive of the community structure of other body regions on the same individual [47]. An individual’s microbiome community structure is also dependent on the environment and the people, animals, and surfaces with which they interact [48]. However, observing that there is a correlation between microbiome community structure and human health does not identify the underlying molecular mechanisms driving this relationship.

**Fig. 1:**
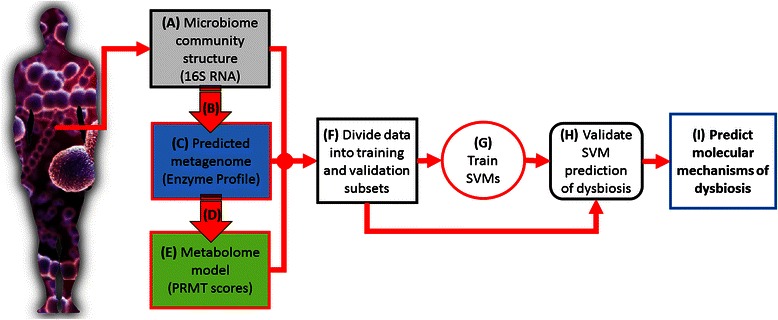
Outline of experimental design. (*A*) 16S rRNA microbiome data, previously reported by David et al. [49], followed the microbiome community structures of two human donors over the course of a year at nearly daily intervals. Microbiome samples can be grouped into dysbiotic states and non-dysbiotic states from observed shifts in microbiome community structures, and knows changes in donors’ health and activities. Using collected sequences and annotated bacterial genomes (*B*), metagenomic enzyme profiles were predicted from reported 16S rRNA community structures (*C*). Using the predicted relative metabolic turnover (PRMT) method (*D*), metabolic models were generated from enzyme function profiles (*E*). All three data types (*A*, *C*, and *E*) were divided into training and validation subsets (*F*). Two approaches were used to divide data into training and validation subsets. The first combined data from donors and selected training and validation subsets to contain an approximately equal number of samples from each donor. In the second approach, training data were selected from a subset of one donor, and all data from the alternate donor were used for the validation set. (*G*) Support vector machines (SVMs) were used to build predictive models from training data sets for each data type. Models predicted whether samples were collected from a donor with a non-dysbiotic or dysbiotic state. (*H*) SVM models were validated on data subsets selected in (*F*). Using features identified as highly predictive for dysbiosis in validated SVM from (*G*), the molecular mechanisms underlying dysbiosis can be proposed (*I*)

We hypothesize that the dysbiotic state of the human-associated gut bacterial community is not caused by the presence or relative abundance of individual bacterial species, but that dysbiosis is an emergent property of the metabolome of the entire microbiome community. A highly relevant, longitudinal study of a microbiome dynamics dataset from a recent study by David et al. [49] was used to test this hypothesis, using the analysis approach outlined in Fig. 1. From the observed microbiome community structures, and using a previously published methodology for inferring metabolomic data from microbial community structures [50], we predicted the metagenomes of microbiomes, expressed as community enzyme function profiles. From the predicted enzyme function profiles, we generated models of community metabolomes (similar to approach used in [51]) . Support vector machines (SVMs) were trained to predict host status, dysbiotic or non-dysbiotic, using one of four possible microbiome feature types: observed microbiome community structures, predicted community enzyme function profiles, and modeled total and secondary community metabolomes. Given a set of training microbiomes, with each microbiome marked as belonging to one of two categories, non-dysbiotic or dysbiotic, an SVM training algorithm builds a model that assigns new microbiomes into one category or the other. This approach has the advantage of not only generating a model capable of predicting dysbiosis from microbiome data, but also identifying the specific enzyme activities or metabolites that can serve as molecular targets for human host therapeutic interventions, or as metabolic markers for human health diagnostics.

## Data description

In a recent longitudinal microbiome study by David et al. [49], two volunteers, identified as Donor A and Donor B, collected stool samples on an approximately daily basis for one year in order to track the dynamics of their respective gut microbial communities. This data set is unique among microbiome studies in that it follows the same, healthy individuals over time; observing their microbiomes before a perturbation and following the recovery of the microbiomes after the disturbance has passed. We used the data generated by this study in our analysis. In the David et al. study, it was observed that gut microbiome community structures for an individual host are generally stable over time, although the microbiomes of the two donors were found to differ significantly from one another. Perturbations to the hosts, however, were found to drive the gut microbiome into a dysbiotic state. During the course of the study, both donors experienced perturbations that profoundly altered their microbiomes: Donor A traveled abroad for an extended period, and Donor B suffered from an intestinal illness. In both cases, after the perturbation the dysbiotic microbiomes returned to a stable, non-dysbiotic structure, although in the case of Donor B, the post-illness microbiome community structures were significantly different from the pre-illness ones, as several bacterial phyla had been driven to extinction during the period of illness. The days for which the microbiomes were in a dysbiotic state are greatly outnumbered by the days for which the microbiomes were in a non-dysbiotic state. The microbiome of Donor A was dysbiotic for 37 days, while that of Donor B was dysbiotic for 7 days. The data from these experiments were generously made available by the authors, providing bacterial taxonomy at the genera level.

Of the 442 bacterial genera reported as detected in the data, only the top 81 most abundant genera, accounting for more than 99.5 % of total microbiome populations by normalized operational taxonomic unit (OTU) counts, were selected for use in the subsequent analyses. The low-abundance, rarely observed taxa making up the lower 0.5 % of the population were disregarded as having a negligible effect on community enzyme profile and metabolome, as previously done using similar methods [50–52]. For each observation, microbiome population abundances were normalized to sum to 100. All microbiome community structure data are available as Additional file 1.

## Analyses

In this study, we used microbiome community structure data to infer the possible enzymatic and metabolic molecular mechanisms underlying dysbiosis. The overall analysis approach is summarized in Fig. 1.

### Microbiome community structures vary by donor and by host dysbiosis state

**Fig. 2:**
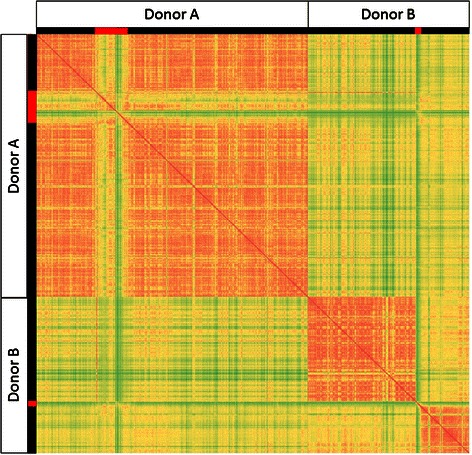
Bray-Curtis dissimilarity indices between all microbiome community structures. BC indices between all pairs of metagenomic samples are indicated for Donor A and Donor B. Samples identified as dysbiotic are indicated in *red* in *left* and *top* borders. Colors in heat map are relative to BC index, with *red* indicating higher BC indices, *green lower* indices, and *yellow* intermediate values. The minimum BC index in the matrix is 54

To quantify how microbiome communities differ by individual (Donor A and B) and by host dysbiosis state (before dysbiosis, dysbiosis, and after dysbiosis), the Bray-Curtis (BC) dissimilarity index was calculated and visualized between all pairs of microbiome samples (Fig. 2). The BC dissimilarity index [53] compares two microbiomes and quantifies the differences between them. A BC index equal to 100 indicates perfect similarity in species identity and abundance between two microbiomes, and a BC index equal to 0 indicates that there are no species in common between the microbiomes. In the matrix of BC scores, it can be seen that similarity within a donor’s samples is higher than similarity between donors. For Donor B, the change in community structure after dysbiosis can also be seen.

### Predicting enzyme function profiles and generating metabolome models from microbiome community structures

**Fig. 3:**
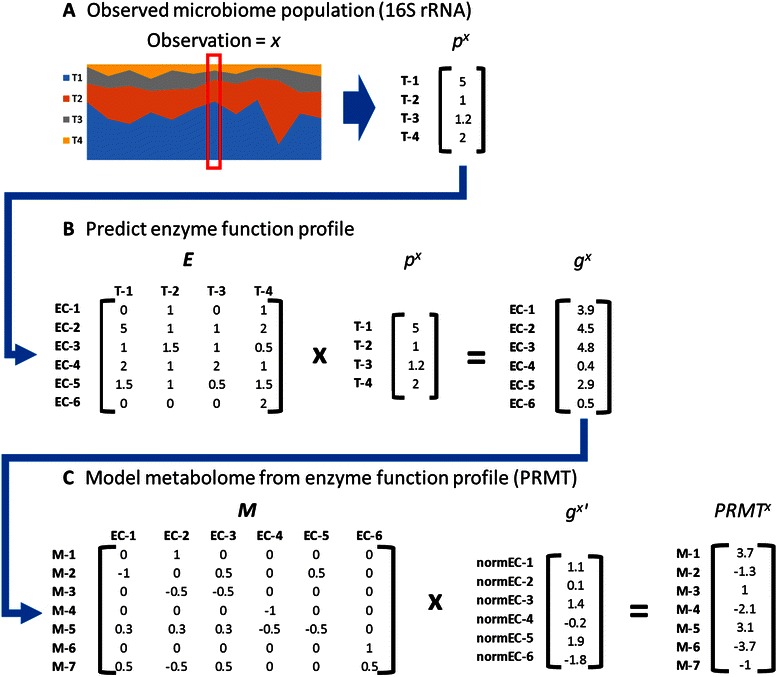
Outline of enzyme function profile prediction and metabolome modeling from microbiome community data. In **a**, data from multiple observations from the microbiome are collected in the form of 16S rRNA abundances. For each observation in each dataset, where a single observation is denoted in the cartoon by red box, the microbiome population is described as a vector of normalized bacterial abundances, *p*. In this cartoon example, the microbiome is composed of four taxa, T 1–4. In **b**, the microbiome population is used to predict the enzyme function profile using a matrix of average enzyme function counts for all bacterial taxa, *E*. Matrix *E* is generated from analysis of published and annotated bacterial genomes. In this cartoon, there are six possible enzyme functions, EC 1–6. In the matrix presented, for example, the average genome of taxa 1 contains two genes annotated with enzyme function EC-4. The result of this step is a matrix for the microbiome’s enzyme function profile, *g*. In **c**, the normalized enzyme function profile *g’* is used to calculate a model of the community metabolome as a vector of PRMT scores. This uses an interaction matrix *M* of enzyme functions and metabolites. In the cartoon example, *M* is comprised of the six enzyme activities in *g* and seven possible metabolites, *m* 1–7. Matrix *M* is generated from available databases of all possible bacterial metabolic reactions for all enzyme activities found in enzyme function profile

Using 16S rRNA metagenomic data and computational approaches that have been previously presented [50, 51], it is possible to extrapolate metagenomic and metabolomic features of the microbiome community (Fig. 3). From taxonomic relative abundance (i.e. community structure) data (Fig. 3a) and a taxonomic average enzyme function count matrix (Fig. 3b), community enzyme function profiles can be extrapolated [50]. The average enzyme function count matrix contains the average number of genes annotated with a specific enzyme function in all annotated genomes for a given bacterial taxon. The community enzyme function profile for a particular microbiome sample is defined as the relative abundance of genes that code for specific enzyme functions in a microbial community’s metagenome. From the community enzyme function profile, the community metabolome, which is defined as the complete set of possible metabolic reactions that can occur in a bacterial community, can be modeled (Fig. 3c). Note that in this definition, the presence and relative abundance of a particular enzyme function indicates that the capacity for a particular metabolic reaction is present in the community, but cannot determine with any certainty that the reaction is actually occurring. The community metabolome was modeled using the predicted relative metabolic turnover (PRMT) scoring metric (Fig. 3c) [51]. PRMT is a computational analysis tool that uses the changing relative abundance of functional genes in metagenomic data between samples to predict the changing capacity of that community to consume or generate metabolites. The community secondary metabolome is a subset of the community metabolome from which core metabolic pathways (e.g. the citrate cycle, glycolysis/gluconeogenesis, fatty acid metabolism, biosynthesis of amino acids, and carbohydrate metabolism) have been removed.

### Enzyme function profiles and metabolic models are better characteristics than community structure to distinguish dysbiotic samples from non-dysbiotic samples

Two methods were used to determine how well dysbiotic samples are distinguished from non-dysbiotic samples for multiple possible data types: multidimensional scaling (MDS) plots and BC dissimilarity indices. These approaches are complimentary. While MDS plots, based on Euclidian distances, globally visualize how similar samples are within a potentially very large dataset, BC indices [53] provide a quantifiable metric for similarity between specific pairs of samples.

#### Multidimensional scaling plots

**Fig. 4:**
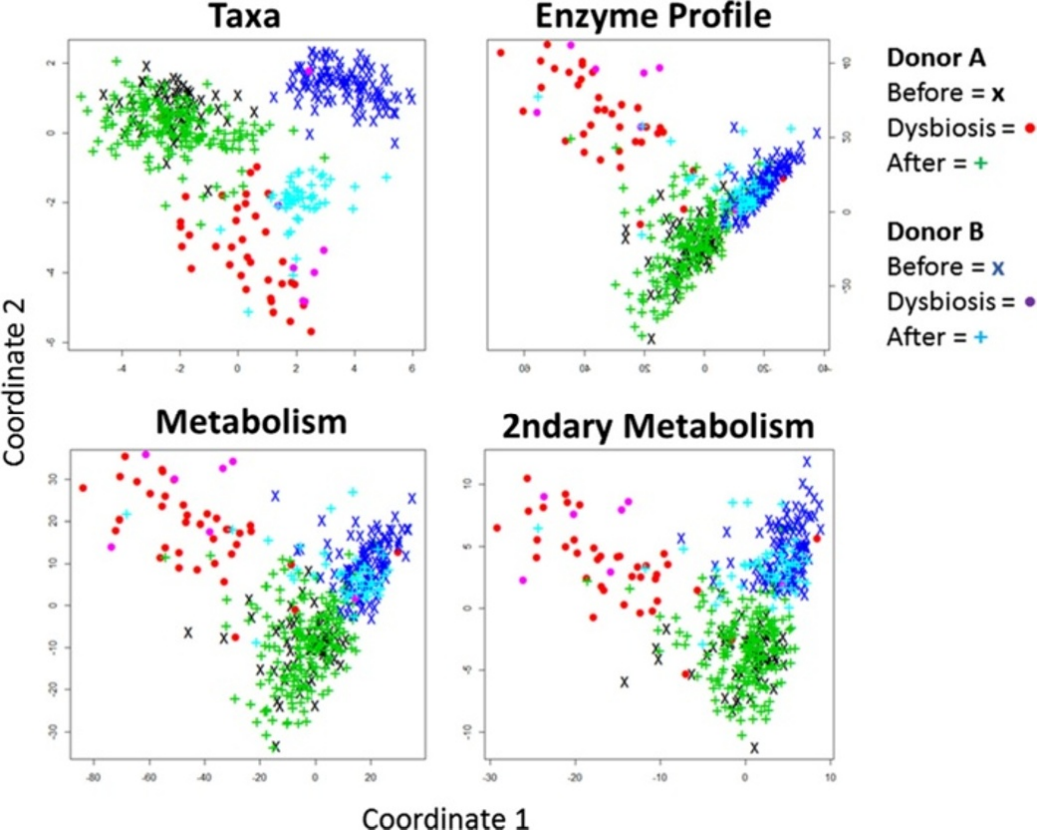
Multidimensional scaling plots for microbiome feature data types. In multidimensional scaling (MDS) plots, each point represents one microbiome sample for two donors (Donors A and B) and three conditions (before dysbiosis, dysbiosis, and after dysbiosis). Four microbiome data features are considered: taxonomic population structures (Taxa), community enzyme function profiles (Enzyme Profile), community total metabolome (Metabolism), and community secondary metabolome (2ndary Metabolism). Points that cluster nearer to one another in an MDS plot are more similar to one another

The MDS plots for taxonomy, community enzyme function profiles, and total and secondary community metabolome demonstrate that the four types of microbiome feature data group donors, and donor microbiomes cluster differently (Fig. 4). When plotted by taxonomic community structure, then donor appears as the microbiome’s most distinguishing characteristic. The microbiomes of Donor A and Donor B group separately and Donor B’s post-illness microbiome groups more closely to the dysbiotic microbiomes than to Donor B’s microbiome pre-illness. When grouped by enzyme profile or by metabolome, then the most distinguishing characteristic of microbiomes becomes donor microbiome state: non-dysbiotic or dysbiotic. Non-dysbiotic microbiomes cluster closest, with the most overlap in total community metabolome.

These results support the hypothesis that dysbiosis of the microbiome is best described as an emergent property of the community metabolome, and is less dependent on the presence or absence of specific bacteria. While community structure alone is not enough to reliably cluster non-dysbiotic from dysbiotic, metabolome can do this. For example, the pre- and post-illness microbiomes for Donor B are very distinct when plotted by community structure **(**Fig. 4). When clustered by metabolome, pre and post-illness communities are more similar. This suggests that there may be a characteristic metabolome for particular human health states, and that a specific metabolome may be assembled by many possible individual microbiome community structures.

#### Bray-Curtis dissimilarity indices

**Fig. 5:**
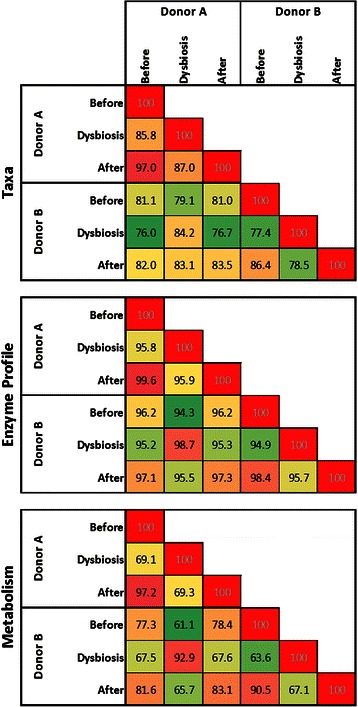
Bray-Curtis dissimilarity between average bacterial populations, grouped by donor and dysbiotic state. Sample data from community structure, enzyme function profile, and community metabolic model were averaged, and grouped by donor and by dysbiosis status. BC indices between all pairs of averaged communities for each data type are presented. Colors in heat map are relative to BC index, with *red* indicating higher BC indices, *green lower* indices, and *yellow* intermediate values

From David et al.’s previously reported investigation of these data [49], as well as from analysis of Fig. 2, it is observed that non-dysbiotic microbiome community structures are stable, but fluctuate substantially when the host experiences a significant perturbation. After perturbation, they then resume a steady state that is potentially novel. We calculated BC dissimilarity indices [53] between the average taxonomic community structure, community enzyme function profile, and community metabolome for Donors A and B for the following host states: before dysbiosis, dysbiosis, and after dysbiosis (Fig. 5).

By BC dissimilarity, dysbiotic samples are always more similar than non-dysbiotic samples across donors and for all data types. For Donor A, pre and post-dysbiosis states are always most similar to one another for all data types. For both enzyme function profile and metabolic model, dysbiotic samples are more similar across donors than dysbiotic and non-dysbiotic within the same donor. While similarity between dysbiotic samples is higher in enzyme function profile than metabolic model, the difference in similarity between cross-donor dysbiotic and cross-donor non-dysbiotic is greater for metabolic models than for enzyme function profiles. These results indicate that there is similarity between dysbiotic samples across donors, and that similarity is enhanced when considering predicted enzyme profiles or metabolic models as opposed to considering community structure data.

### Predicting dysbiosis from microbiome features

SVMs were generated to predict dysbiotic state from microbiome features. Two approaches to training and validating SVMs were taken. For the first approach, all donor data were combined and the training data set was drawn equally for Donors A and B from both dysbiotic and non-dysbiotic samples. The validation data set was the remaining Donor A and B data not used in the training set. In the second approach, the highly predictive features identified by combined microbiome data were used in a cross-donor validation experiment. In the cross-donor experiment, training data were drawn entirely from one donor and the resulting SVMs were validated on the entire dataset from the other donor. The cross-donor approach also removes the possibility of over-fitting by SVM. For both methods, prediction accuracy on validation sets is presented as an F-score, a combination of precision and recall of a SVM model.

#### SVM trained with the combined donor microbiome data are strongly predictive of host dysbiosis for all microbiome feature types

The randomly selected training set for the combined donor data is comprised of 60 non-dysbiotic samples and 20 dysbiotic samples. Non-dysbiotic samples are equally composed of 15 microbiomes each from Donor A and Donor B, pre and post-dysbiotic samples. The dysbiotic training set is comprised of 15 dysbiotic samples from Donor A and five dysbiotic samples from Donor B. The validation set is the remaining data, comprised of 375 non-dysbiotic samples and 22 dysbiotic samples.

As ranked by Fisher score, SVMs were trained on the top scored 100, 90, 80, 70, 60, 50, 40, 30, 20, and 10 % of features (i.e. taxonomic community structure, community enzyme function profile, total community metabolome, and secondary community metabolome). For SVM models with enzyme function profile and total metabolome, the features were further divided into sets of the top 5, 2.5, 1.25, and 0.625 % ranked by Fisher score. The smallest subset for all feature types was about 10 features.

**Fig. 6:**
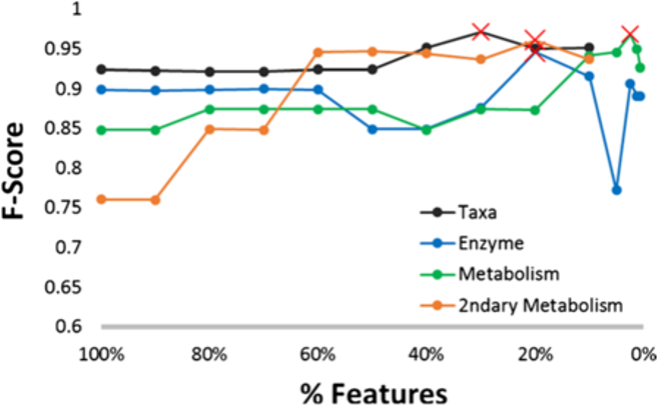
Predicting host status on four types of microbiome information: combined donor results. Each point on the graph shows the results of an SVM trained on a subset of community structure, enzyme function profile, and community total and secondary metabolism. The X-axis is the percent of features, selected from top-ranked Fisher score, used to train SVMs. Y-axis is the F-score for the prediction accuracy of the SVM model. Red ‘Xs’ identify the training data subsets that produced the most predictive models

All SVMs yielded good, predictive models for identifying dysbiotic samples from microbiome feature data (Fig. 6). When trained on data combined from both donors, SVM performs well using all microbiome features: taxonomic community structure (best F-score 0.97), community enzyme function profile (best F-score 0.95), total community metabolome model (best F-score 0.97), and secondary metabolome (best F-score 0.96).

Combined donor analysis does not well support the hypothesis that microbiome community function is best described as an emergent property of community structure. All microbiome data types are found to be roughly equivalently useful for predicting dysbiosis. To look deeper into this dataset and seek potentially biologically relevant molecular mechanisms of dysbiosis, we turn to the more stringent cross-donor analysis.

#### SVM trained using cross-donor validation demonstrates significant differences between the predictive powers of different microbiome feature types

A significant challenge in microbiome analysis is that it is difficult to apply results across individuals given the inherent variation between individual microbiomes. To address this, we have chosen a validation scheme to train SVM models on only one donor, and then validate on the other. A model that is successful in identifying patterns spanning individual variation can be more confidently assumed to have identified underlying biological principles in microbiome–host interactions, and not microbiome characteristics that may be unique to a specific individual.

The set of microbiome features used in the cross-donor analysis is taken from the most predictive feature subsets from the previous results, based on the combined Donor A and B data (Fig. 6). The most predictive subsets identified in the combined donor data are 24 genera, 380 unique enzyme functions, 36 metabolites from total community metabolome, and 24 secondary metabolites from secondary community metabolome. For the SVM trained on Donor A and validated on Donor B, there are 30 randomly selected non-dysbiotic samples and 12 dysbiotic samples. For the SVM trained on Donor B and validated on Donor A, there are 30 randomly selected non-dysbiotic samples and 7 dysbiotic samples. Validations were performed on the entire set of alternate donor data.

**Fig. 7:**
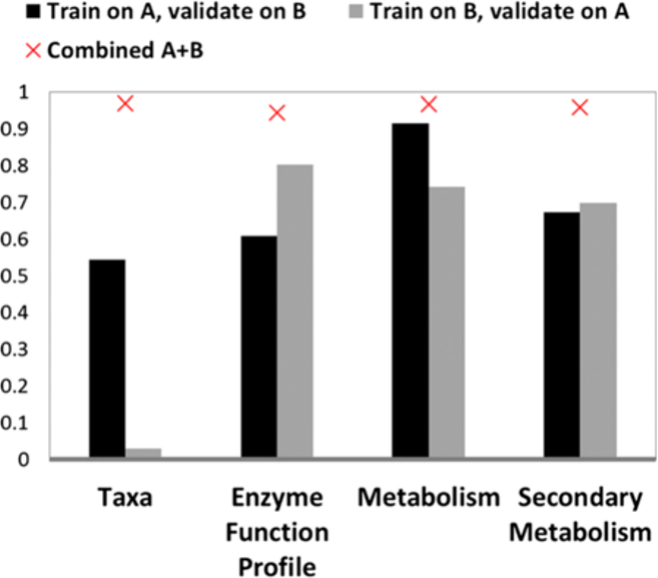
Predicting host status on four types of microbiome information: cross-donor validation results. F-scores for cross-donor SVM predictions are given by *black* (model trained on Donor A data and validated on donor B data), and *gray* (model trained on Donor B data and validated on Donor A data) bars. F-scores for SVM trained on mixed-model data are displayed as red ‘Xs’; values were taken from the most predictive SVM parameters and training sets identified from Fig. 7

Unlike the results for the combined donor data, cross-donor validated SVM results differed significantly by microbiome feature types (Fig. 7). In the cross-donor validated SVM, microbiome community structure is the least predictive, with SVM trained on Donor B data and validated on Donor A data performing very poorly (F-scores 0.545 and 0.03 for Donor A and Donor B training sets respectively). Microbiome total community metabolome feature data performs best in the cross-donor validation (F-scores 0.92 and 0.74), with results for community enzyme function profile (best F-scores 0.61 and 0.83) and secondary community metabolome (F-scores 0.67 and 0.70) roughly equivalent to one another.

Cross-donor analysis supports the hypothesis that microbiome community function is an emergent property of community structure. Community metabolome is much more predictive of dysbiosis than the underlying microbiome community structure.

### Highly predictive features identified by SVM provide insights into molecular mechanisms of dysbiosis

In the previous sections, microbiome features have been demonstrated to be predictive of dysbiotic states. While this provides evidence that analysis of the microbiome might be diagnostic for host health, it does not provide the information required to suggest the mechanisms by which microbiome is predictive of host dysbiosis, or propose possible interventions by which the microbiome could be successfully manipulated to influence host health. To investigate possible molecular mechanisms by which microbiome activity and host health may be related, we consider the metabolic pathways that are statistically significantly enriched for the sets of genera (Table 1), community enzyme function profile (Table 2), total community metabolome (Table 3) and secondary community metabolome (Table 4).

**Table 1:** Bacterial genera most predictive of dysbiosis

Phylum	Class	Order	Family	Genus
Bacteroidetes	Bacteroidia	Bacteroidales	Porphyromonadaceae	**Parabacteroides**
Firmicutes	Bacilli	Gemellales	Gemellaceae	**Gemella**
		Lactobacillales	Carnobacteriaceae	**Granulicatella**
			Enterococcaceae	**Enterococcus**
			Streptococcaceae	**Lactococcus**
	Clostridia	Clostridiales	Clostridiaceae	**Clostridium**
			Lachnospiraceae	Coprococcus
				**Epulopiscium**
				Lachnobacterium
				Roseburia
			Veillonellaceae	**Veillonella**
Fusobacteria	Fusobacteria	Fusobacteriales	Fusobacteriaceae	**Fusobacterium**
Proteobacteria	Epsilonproteobacteria	Campylobacterales	Campylobacteraceae	**Campylobacter**
	Gammaproteobacteria^a^	Enterobacteriales^a^	Enterobacteriaceae^a^	**Enterobacter**
				**Erwinia**
				**Escherichia**
				**Klebsiella**
				**Morganella**
				**Pantoea**
				**Plesiomonas**
				**Serratia**
				**Tatumella**
				**Trabulsiella**
		Pasteurellales	Pasteurellaceae	**Haemophilus**

Taxonomies (class, order, or family) that are significantly enriched (*p*-value <0.05) in the set of genera highly predictive of dysbiosis, relative to all genera found to be present in microbiomes, are identified with ‘^a^’. Genera that are more abundant in dysbiotic microbiomes are highlighted with in bold text

**Table 2:** Enriched pathways in most predictive community enzyme function profile features

KEGG ID	Pathway	Unique enzyme function	*p*-val
map00121	Secondary bile acid biosynthesis	1.-.-.- | 4.2.1.- | 6.-.-.-	0.00
map01053	Biosynthesis of siderophore group nonribosomal peptides	1.3.1.28 | 3.3.2.1 | 2.7.7.58 | 6.3.2.-	1.08×10^−2^
map00540	Lipopolysaccharide biosynthesis	2.4.1.56 | 2.4.-.- | 3.6.1.- | 2.7.1.- | 5.1.3.20 | 3.1.3.- | 5.-.-.- | 2.3.1.- | 6.-.-.- | 2.4.1.44	1.65×10^−2^
map00904	Diterpenoid biosynthesis	1.14.11.- | 1.14.13.- | 2.3.1.-	3.06×10^−2^
map00053	Ascorbate and aldarate metabolism	4.2.1.42 | 4.1.1.85 | 4.2.1.40 | 3.1.1.- | 4.1.2.20 | 3.7.1.- | 5.1.3.22 | 1.1.1.122 | 1.1.1.130 | 3.1.3.- | 5.1.3.4 | 2.7.1.53	3.45×10^−2^
map00480	Glutathione metabolism	3.5.1.78 | 3.4.11.23 | 4.1.1.17 | 6.3.2.3 | 1.8.1.7 | 1.17.4.1 | 2.5.1.18 | 2.3.2.2 | 6.3.1.8 | 3.5.2.9	3.76×10^−2^
map00906	Carotenoid biosynthesis	1.-.-.- | 2.5.1.- | 1.14.13.- | 5.-.-.- | 2.3.1.-	4.68×10^−2^

**Table 3:** Enriched pathways in most predictive total community metabolome model features

KEGG ID	Pathway	Metabolites	*p*-val
map00770	Pantothenate and CoA biosynthesis	CoA | Pantetheine_4′-phosphate | Apo-_acyl-carrier-protein_	2.86×10^−4^
map00561	Glycerolipid metabolism	Phosphatidate | Diglucosyl-diacylglycerol | Glycerophosphoglycoglycerolipid	5.16×10^−4^
map00030	Pentose phosphate pathway	5-Phospho-alpha-D-ribose_1-diphosphate | D-Ribose_1,5-bisphosphate | 2-Dehydro-3-deoxy-6-phospho-D-gluconate	6.71×10^−4^
map00361	Chlorocyclohexane and chlorobenzene degradation	2-Maleylacetate | 2,4-Dichlorophenol | cis-2-Chloro-4-carboxymethylenebut-2-en-1,4-olide | 2-Chloromaleylacetate	2.57×10^−3^
map00240	Pyrimidine metabolism	| 5-Phospho-alpha-D-ribose_1-diphosphate | Thymine	4.72×10^−3^
map00362	Benzoate degradation	2,3-Dihydroxybenzoate | S-Benzoate_coenzyme_A | 2-Maleylacetate	6.56×10^−3^
map00627	Aminobenzoate degradation	2,3-Dihydroxybenzoate | S-Benzoate_coenzyme_A | 2-Maleylacetate	6.56×10^−3^
map01120	Microbial metabolism in diverse environments	5-Phospho-alpha-D-ribose_1-diphosphate | 2,3-Dihydroxybenzoate | S-Benzoate_coenzyme_A | 2-Maleylacetate | 2,4-Dichlorophenol | 5,10-Methenyltetrahydromethanopterin | 5,10-Methylenetetrahydromethanopterin | 2-Dehydro-3-deoxy-6-phospho-D-gluconate | cis-2-Chloro-4-carboxymethylenebut-2-en-1,4-olide | Aerobactin | Ectoine | 2-Chloromaleylacetate | 2-Hydroxy-cis-hex-2,4-dienoate | 4-Fluoromuconolactone | 2-Chloro-5-methylmaleylacetate	1.57×10^−2^

**Table 4:** Enriched pathways in most predictive secondary community metabolome model features

KEGG ID	Pathway	Secondary metabolites	*p*-val
map01061	Biosynthesis of phenylpropanoids	L-Tryptophan | p-Coumaroyl-CoA | Coniferyl_alcohol | 4-Coumarate | Caffeate | Ferulate | Coniferyl_aldehyde | 4-Hydroxycinnamyl_aldehyde | 5-Hydroxyferulate | 5-Hydroxyconiferaldehyde	7.93×10^−7^
map01120	Microbial metabolism in diverse environments	5-Phospho-alpha-D-ribose_1-diphosphate | 2,3-Dihydroxybenzoate | S-Benzoate_coenzyme_A | 2-Maleylacetate | 2,4-Dichlorophenol | 5,10-Methenyltetrahydromethanopterin | 5,10-Methylenetetrahydromethanopterin | 2-Dehydro-3-deoxy-6-phospho-D-gluconate | cis-2-Chloro-4-carboxymethylenebut-2-en-1,4-olide | Aerobactin | Ectoine | 2-Chloromaleylacetate | 2-Hydroxy-cis-hex-2,4-dienoate | 4-Fluoromuconolactone | 2-Chloro-5-methylmaleylacetate	1.57×10^−2^
map00940	Phenylpropanoid biosynthesis	p-Coumaroyl-CoA | Coniferyl_alcohol | 4-Coumarate | Caffeate | Ferulate | Coniferyl_aldehyde | 4-Hydroxycinnamyl_aldehyde | 5-Hydroxyferulate | 5-Hydroxyconiferaldehyde | 5-Hydroxyconiferyl_alcohol | N1,N5,N10-Tri-_hydroxyferuloyl_-spermidine	1.47×10^−6^
map04974	Protein digestion and absorption	L-Tryptophan | L-Leucine | Tyramine	1.26×10^−2^

While not definitive without additional biological experimental confirmation, these pathways and metabolites are strong candidates for hypothesis-driven biological experiments to deepen understanding of the relationship between human health and its symbiotic microbiome.

#### Dysbiosis leads to changes in microbiome vitamin metabolism

One important function of the gut microbiome is the biosynthesis of vitamins that are important to the host [1, 38]. Affected pathways “Pantothenate and Co biosynthesis” (vitamin B) (Table 3), “Ascorbate and aldarate metabolism” (vitamin C) (Table 2), and “Carotenoid biosynthesis” (antioxidants) (Table 2) indicate that dysbiosis may interfere with the microbiome’s ability to provide these vitamins to its host.

#### Dysbiosis affects host’s digestion

Protein degradation and digestion are affected in dysbiosis, as indicated by the enrichment of pathways “Biosynthesis of phenylpropanoids”, “Phenylpropanoid biosynthesis” [54] (Table 4), and “Protein digestion and absorption” (Table 4). Amines such as putrescine and spermidine (Table 4) are also associated with the breakdown of proteins [55]. Changes in fatty acid digestion and absorption are suggested by enrichment for the pathways “Glycerolipid metabolism” (Table 3) and “Secondary bile acid biosynthesis” (Table 2). Secondary bile acids are those resulting from bacterial metabolism in the gut. These results suggest that dysbiosis changes the way in which the host digests and absorbs food.

#### Virulence factors in the dysbiotic microbiome

Both the iron-scavenging metabolite aerobactin (Table 3) and the enriched pathway for “Biosynthesis of siderophore group nonribosomal peptides” (Table 2) can be virulence factors [56, 57], and both are predictive of a dysbiotic gut microbiome. *Enterobacteriacae* are statistically enriched and found in increased abundance in the set of predictive genera relative to the complete set of bacterial species identified in the complete gut microbiome communities (Table 1). *Enterobacteriacae* includes potential pathogen species in the genera *Enterobacter*, *Klebsiella*, and *Plesiomonas*. While not directly associated with virulence, the pathways “Aminobenzoate degradation “and “Benzoate degradation” (Table 3) are implicated in IBS [58, 59].

### Prediction of community enzyme function profile and metabolome is robust against the effects of possible annotation errors in bacterial genomes

A significant concern of predicting community metagenomes and metabolomes from community structure data is the presence of possible annotation errors present in the body of sequenced and annotated bacteria genomes. In this case, ‘errors’ at the level of annotated genomes might be due to erroneous or missing gene annotations. At the taxonomic level of genera, the average enzyme function abundance for a specific enzyme’s activity might be biased towards the specific distribution of sequenced organisms, and not necessarily representative of the distribution of organisms present in the microbiome. While improving the accuracy of the available collection of annotated bacterial genomes is beyond the scope of this work, we estimated the possible effect of erroneous gene annotations by adding random noise to the gene function counts in the bacterial genomes used to predict community enzyme function profiles.

**Fig. 8:**
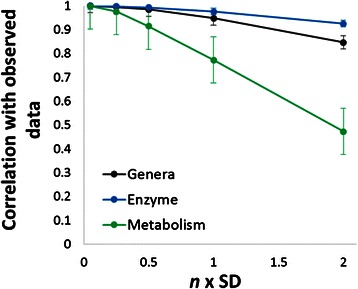
Determining the effect of gene annotation errors on the prediction of community enzyme function profile and community metabolism. On the X-axis, the amount of noise added to genera-level average enzyme function counts is given as a factor of *n* standard deviations. Y-axis is the Pearson’s correlation coefficient between the noise-added dataset and original data. Error bars are ± one standard deviation from five experimental replications

Random noise was added to genera average enzyme function counts as a multiple *n* of the standard deviation of enzyme function counts across all sequenced bacterial genomes. Noisy genera-level average enzyme function counts were generated for *n* equal to 0.05, 0.25, 0.5, 1, and 2, with five replicates each *n* for a total of 25 noise-added genera-level average enzyme function counts. The noise-added enzyme function counts were used to generate predicted community enzyme function profiles and metabolic models as described above for a total of 298,750 samples each of noise-added predicted enzyme function profiles and PRMT-score-based metabolic models. The Pearson’s correlation coefficients between matrices for noise-added samples and initial data were calculated (Fig. 8).

Predicted community enzyme function profiles were found to be less vulnerable to random noise than the genera-level enzyme function profiles for annotated genomes. Community metabolome models are most sensitive to the addition of random noise. Both noise-added predicted community enzyme function profiles and metabolic models correlate with the initial observation with a correlation greater than 0.9, even with a noise of ± 0.5 SD to every enzyme function count for every genus. While it is impossible to say with certainty to what degree the predicted enzyme function profiles or metabolic models accurately reflect the true biological states of the microbiome communities, it is evident from this analysis that those predictions are stable to substantial variations in the set of annotated genomes. We can be confident that our analysis will address our desired hypothesis, and is not likely to be skewed by quirks of the available database of sequenced organisms.

## Discussion

The microbiome community and its human host are intimately bound together in symbiosis. Actions of the host can affect the microbiome community, and in turn, the microbiome community has a powerful influence on host health. In a recent longitudinal study, the microbiome of two donor volunteers was tracked over the course of a year. Microbiome community structures were observed to be in one of two possible states: non-dysbiotic or dysbiotic. After a perturbation, community structure quickly became stabilized to a non-dysbiotic state. Using metabolic modeling with SVM, we have identified the characteristic metabolomes of these two states, and have shown that these states are less dependent upon specific host or particular microbiome community structure. Rather, they are better described as an emergent property of the microbiome and its aggregate community metabolome.

When data from Donors A and B are combined, there is very little difference in the predictive capacity of community structure data, predicted enzyme function profile, or metabolic model. However, when the far more challenging cross-donor validation is attempted, strong differences become apparent between the predictive powers of different feature types. The ability of community structure feature data to predict dysbiosis drops precipitously on the cross-donor validation scheme. In particular, when the SVMs are trained on data from Donor B, the ability to predict dysbiosis in Donor A is worse than random. Feature types of community enzyme function profile and metabolic model, however, are able to effectively predict dysbiosis, even in the cross-donor validation scheme. Total metabolome model has a slight advantage over enzyme function profile and secondary metabolic model in the cross-donor validation.

The most significant advantage of SVM trained on metabolic model feature types, however, is not a better ability to predict dysbiosis, but rather the ability of metabolic models to propose possible molecular interactions that drive dysbiosis, although biological validation of these predictions is beyond the scope of this work. Pathways for vitamin biosynthesis [1, 6], protein and fatty acid digestion [54, 55, 60], and potential virulence factors [56–59] were found to be significantly enriched for the predictive microbiome features. These features propose specific mechanisms of microbiome–host interactions that will form the basis of additional, hypothesis-driven biological experiments.

While this analysis successfully demonstrated that, for the cross-donor analysis, emergent properties of microbiome community are more predictive than the community structures themselves, there is much additional work that can be anticipated. While the accuracy of predictions for dysbiosis was strong, it is very possible that a mixed-model SVM might be more predictive than using a single microbiome feature type. However, a mixed-model approach was not useful in the current study, in which the biological hypothesis is that metabolomic model data is more predictive than microbiome population structure. In addition, while it provided an excellent opportunity for demonstrating the potential power of a microbiome metabolome-based predictor of dysbiosis, a predictive model that was constructed on only two otherwise healthy adult donors cannot likely be generalized to the full range of possible host phenotypes and dysbiosis types. We anticipate the opportunity to expand this approach to a wider range of host phenotypes and dysbioses as additional microbiome data becomes available. While prediction of metagenomic data from community structures is a useful tool, further experiments in which the metagenome is directly sequenced and the metabolome is directly observed, are needed to validate computational predictions. Also, while SVM was the predictive tool used here, in future studies where optimizing prediction accuracy is the goal for use in patient diagnostics, additional machine tools such as random forest or logistic regression should be considered. Fortunately, investigations into host–microbiome interactions are becoming more common, providing additional opportunities to study the impact of the microbiome on human health and making analysis approaches like the one we present here an increasingly important tool in driving future experiments.

## Methods

### Predict community enzyme function profiles from community structure

To extrapolate microbiome enzyme function profiles, we followed the protocol outlined in [50], which is summarized here and outlined in Fig. 8b. Enzyme commission (EC) annotations [61] were used for our ontology of possible enzyme functions. The method used here has similarity to the PiCRUST method [62], which generates metagenomic predictions using the closest 16S rRNA similarity to published genomes, and uses an alternative gene function annotation ontology. The enzyme function profile for microbiome *x* is calculated as:

$$ \overrightarrow{g^x}=\overrightarrow{p^x}\boldsymbol{E} $$

Where:

- $\overrightarrow{p^x}$ is a vector denoting microbiome community structure *x*, with length *T*, $\overrightarrow{p^x}=\left\{{p}_1^x,{p}_2^x,\dots {p}_T^x\right\}$, and *T* is the total number of taxa represented in the microbiome.
- ***E*** is a taxonomic average enzyme function count matrix for genomic enzyme function counts of size *EC* x *T*, where *EC* is the number of all possible represented EC annotations for unique enzyme activities, and *T* is the number of all bacterial taxa under consideration. Each entry *E*_*ec,t*_ is the average number of genes with specific annotation *ec* for all genomes of a particular taxa, *t*. This matrix was previously presented in [50].
- $\overrightarrow{g^x}$ is the resulting vector for the enzyme function profile of microbiome *x*, of length EC, $\overrightarrow{g^x}=\left\{{g}_1^x,{g}_2^x\dots {g}_{EC}^x\right\}$.

All predicted microbiome community enzyme function profiles are available in Additional file 2.

### Generate community metabolome models from community enzyme profiles

Using PRMT scores, it is possible to generate a prediction of microbiome meta-metabolome from enzyme function profiles. PRMT is described in [63], outlined in Fig. 8c, and summarized briefly below. PRMT scores are calculated as:

$$ \overrightarrow{PRMT}=\left(\overrightarrow{g^{\mathit{\hbox{'}}x}}-\overrightarrow{g^{\mathit{\hbox{'}}ave}}\right)\boldsymbol{M} $$

Where:

- $\overrightarrow{g^{\mathit{\hbox{'}}x}}$ is the log-transformed vector of enzyme function profile $\overrightarrow{g^x}$ for microbiome *x*, as calculated in the previous section.
- $\overrightarrow{g^{\mathit{\hbox{'}}ave}}$ is the log-transformed vector of the average of all enzyme function profiles for all microbiomes in the experimental set.
- ***M*** is an enzymatic reaction matrix of size *L* x *EC*, where *L* in the number of ligands in all possible enzymatic reactions by the set of *EC* enzyme functions. As described in [44], this matrix is normalized by network topology and not by reaction stoichiometry.
- $\overrightarrow{PRMT}$ is the resulting vector of PRMT scores of length *L*. A positive PRMT score indicates an increased relative capacity for the production of a compound in the metabolome encoded by microbiome *x*, relative to the average of all observed microbiomes. A negative PRMT score indicates an increased relative capacity for the consumption of a compound in the metabolome encoded by microbiome *x*, relative to the average of all observed microbiomes. PRMT scores do not indicate rates of reaction or predict quantities or concentrations of compounds in a metabolome.

Two types of community metabolomes were calculated using the PRMT method: total community metabolome, and secondary community metabolome. Total metabolome PRMT scores used all possible KEGG reaction pathways [54, 55]. Secondary community metabolome PRMT scores restricted metabolic predictions to a subset of secondary metabolism KEGG networks, comprised of pathway KEGG ID numbers 01110, 00940, 00945, 00941, 00944, 00942, 00943, 00901, 00403, 00950, 00960, 01058, 0023, 00965, 00966, 00402, 00311, 00332, 00331, 00521, 00524, 00231, 00401, and 00254. Secondary community metabolome is a subset of total community metabolome.

The complete, predicted community metabolic network (‘*M’* in Fig. 3b) is comprised of 2,830 metabolites connected by 4,285 enzymatic transformations and 1,901 unique enzyme functions, and is available in Additional file 3. In PRMT-based metabolomic predictions, as a consequence of the metabolic network topology in which some enzyme functions interact with multiple possible metabolites, many sets of metabolites in the model share the exact same patterns of PRMT scores across all samples. For example, many metabolites in the fatty acid biosynthesis pathway (KEGG map00061) interact with the same set of enzyme functions, making their relative metabolism identical to one another. Some metabolites have PRMT scores of 0 for all samples. Prior to any subsequent analysis of PRMT scores, all sets of metabolites with identical PRMT scores were combined into a single metabolite name (e.g. Hexanoyl-[acp], Octanoyl-[acp], Decanoyl-[acp], Dodecanoyl-[acp], etc. are indistinguishable by PRMT score, so they are combined under a single metabolite name). All metabolites with PRMT scores always equal to zero were removed. After this consolidation of non-unique metabolites, the number of metabolites in the total community metabolome was reduced from 2,830 metabolites to 1,492, and in the secondary community metabolome from 209 to 122. The complete set of community metabolome model PRMT scores is available in Additional file 4. A graphical network visualization that integrates community metabolic network topology, secondary metabolism, and PRMT score is available in Additional file 5.

### Adding noise to genomic enzyme function counts

For each count of average enzyme function in each genus, random noise was added using the following formula:

$$ EC\_ nois{e}_i^g= MAX\left[0,E{C}_i^g+nS{D}_i\left(2RND-1\right)\right] $$

Where:

• *EC*_*noise*_*i*_^*g*^

is the enzyme function count adjusted by the addition of random noise for enzyme activity *i* in taxonomic group *g*.

• *EC*_*i*_^*g*^

is the observed enzyme function count for activity *i* in taxonomic group *g*.

• *SD*_*i*_

is the standard deviation of enzyme function counts for activity *i* over all annotated bacterial genomes.

• *n*

is a multiplier applied to the standard deviation.

• RND

is a function that returns a random number between 0 and 1.

• MAX

is a function that returns the maximum of two values.

All of the noise-added taxa enzyme function count tables are available in Additional file 6.

### Multidimensional scaling

Multidimensional scaling (MDS) plot is a graphical approach for comparing similar features in highly complex datasets. For generation of MDS plots, R-project (v 3.0.3) was used [64]. MDS plots for microbiome community structures, log-transformed community enzyme profiles, and total and secondary community metabolome models were generated. MDS plots were calculated using Euclidian distances.

### Support vector machines

To test the hypothesis that emergent properties, such as enzyme function profile or metabolome, are more predictive of host dysbiosis, SVMs were used. For generation of an SVM, R-project and package ‘e1071’ v1.6-1 [65] were used. SVMs were trained on training sets using a 10-fold cross-validation procedure and linear kernels based on total accuracy.

SVMs were trained on multiple subsets of data using features selected based on Fisher score. Fisher score for each taxonomic abundance, enzyme function count, or PRMT-scored metabolic feature *i* is calculated as:

$$ Fisher Scor{e}_i=\frac{\left|\mathrm{Average}\left(non\_ dysbioti{c}_i\right)-\mathrm{Average}\left( dysbioti{c}_i\right)\right|}{\mathrm{SdDev}\left(Al{l}_i\right)} $$

Where:

• Average(*non*_*dysbiotic*_*i*_) is the average of all genera abundance, enzyme function profile, or PRMT scores of non-dysbiotic samples for feature *i*.

• Average(*dysbiotic*_*i*_) is the average of all genera abundances, enzyme function profile, or PRMT scores of dysbiotic samples for feature *i*.

• SdDev(*All*_*i*_) is the standard deviation of all genera abundances, enzyme function profile, or PRMT scores for feature *i*.

### Prediction accuracy as F-score

Accuracy of SVM predictions on validation sets were calculated as F-scores; a combination of the precision and recall:

$$ Fscore=2\frac{precision\;*\; recall}{precision+ recall} $$

Where

$$ precision=\frac{true\; positives}{true\; positives+ false\; positives} $$

and

$$ recall=\frac{true\; positives}{true\; positives+ false\; positives} $$

### Enrichment of KEGG pathways

Features highly predictive of dysbiosis are potentially lengthy lists of genera, enzyme functions, or metabolites. To understand how these lists of features relate to a system-scale understanding of metabolism, we identified specific KEGG pathways that are enriched for the sets of predictive features. Enrichment is calculated using the cumulative hypergeometric distribution as:

$$ Enrichment\_KEG{G}^k=1\hbox{-} \mathrm{HypgeoDist}\left(k,n,K,N\right) $$

Where:

• HypgeoDist is the cumulative hypergeometric distribution.

• *k* is the number of enzymes or metabolites identified as highly predictive by SVM and also associated with KEGG pathway *p*. Enrichment is only considered possible if *k* is greater than or equal to 3.

• *n* is the total number of enzymes or metabolites identified as highly predictive by SVM.

• *K* is the number of enzymes or metabolites in the complete metabolic model and also associated with KEGG pathway *p*.

• *N* is the total number of enzymes or metabolites in the complete metabolic model.

• *Enrichment_KEGG*^*k*^ is expressed as a *p*-value. Significance is considered at a *p*-value less than or equal to 0.05.

## Availability of supporting data

All data used in this analysis can be found in the Additional files, as well as archived in the *GigaScience* GigaDB repository [66].

## Additional files

Additional file 1: **All normalized microbiome community structures for Donors A and B, presented at the taxonomic level of genera and comprising at least 99.5 % of the population observed OTU abundances.** Last row in table is Fisher scores, used to identify most predictive features. (TXT 309 kb) Additional file 2: **Community enzyme function profiles, predicted from microbiome community structures.** Last column in table is Fisher scores, used to identify most predictive features. (TXT 16394 kb) Additional file 3: **Metabolomic network for microbiome community metabolome is presented as a list of reaction in the format Reactant, Enzyme Function (as EC annotation), and Product.** (TXT 228 kb) Additional file 4: **All metabolome model predictions, calculated as PRMT scores from community enzyme function profiles, for all microbiome community samples.** Last column in table is Fisher scores, used to identify most predictive features. (ZIP 10034 kb) Additional file 5: **Metabolic network for microbiome community is presented as a graphical network.** In the graphical representation, nodes are metabolites and directed edges are enzyme-mediated metabolite transformations. The sizes of nodes and the widths of edges are proportional to their Fisher score for dysbiotic state compared to non-dysbiotic sate. Nodes highlighted with a green border are in the Secondary Metabolism network. Nodes highlighted in red are the 36 features most predictive by Total Metabolism, and nodes highlighted in blue are the 24 features most predictive by Secondary Metabolism. Network image was generated using ‘Cytoscape’ [67, 68]. Figure was generated using network information in Additional file 3 and data in Additional file 4. (PDF 547 kb) Additional file 6: **All noise-added taxonomic average enzyme function count matrices.** (ZIP 14369 kb)

## Abbreviations

BC: Bray-Curtis
KEGG: Kyoto Encyclopedia on Genes and Genomes
OTU: Operational taxonomic unit
PRMT: Predicted relative metabolic turnover
SVM: Support vector machine
