# Supplementary figures and images for: Metabolome of human gut microbiome is predictive of host dysbiosis
Source: Gigascience. 2015 Sep 14;4:42. doi: 10.1186/s13742-015-0084-3 (PMC4570295; doi:10.1186/s13742-015-0084-3)

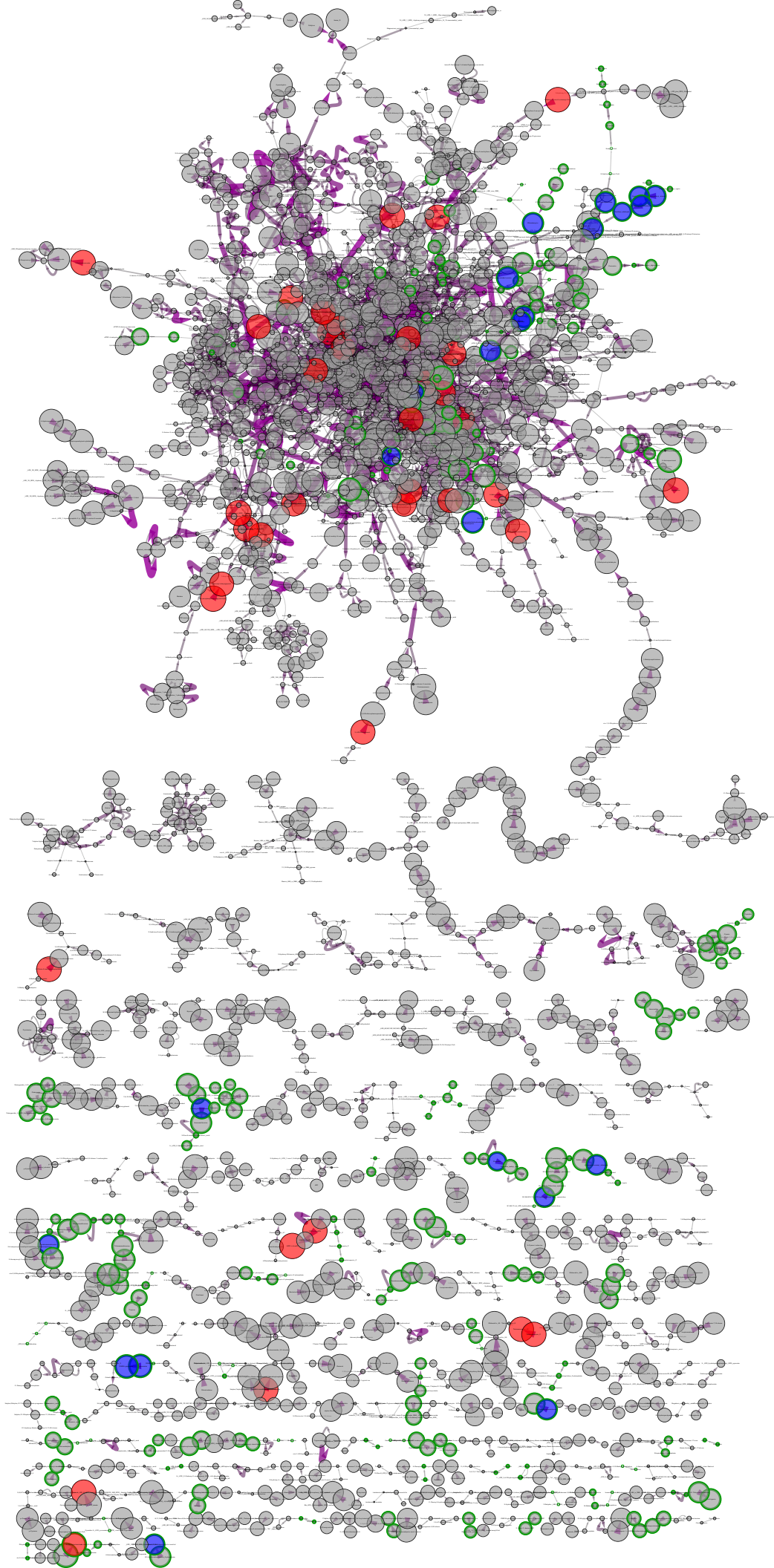

Supplement: Additional file 5: — Metabolic network for microbiome community is presented as a graphical network. In the graphical representation, nodes are metabolites and directed edges are enzyme-mediated metabolite transformations. The sizes of nodes and the widths of edges are proportional to their Fisher score for dysbiotic state compared to non-dysbiotic sate. Nodes highlighted with a green border are in the Secondary Metabolism network. Nodes highlighted in red are the 36 features most predictive by Total Metabolism, and nodes highlighted in blue are the 24 features most predictive by Secondary Metabolism. Network image was generated using ‘Cytoscape’ [67, 68]. Figure was generated using network information in Additional file 3 and data in Additional file 4. (PDF 547 kb) [file 13742_2015_84_MOESM5_ESM.pdf]
